# Glutathione-sensitive nanoparticles enhance the combined therapeutic effect of checkpoint kinase 1 inhibitor and cisplatin in prostate cancer

**DOI:** 10.1063/5.0126095

**Published:** 2022-11-21

**Authors:** Shirong Peng, Xinyu Zhang, Hao Huang, Bisheng Cheng, Zhi Xiong, Tao Du, Jun Wu, Hai Huang

**Affiliations:** 1 Department of Urology, Sun Yat-Sen Memorial Hospital, Sun Yat-Sen University, 107. W. Yanjiang Road, Guangzhou 510220, China; 2 Guangdong Provincial Key Laboratory of Malignant Tumor Epigenetics and Gene Regulation, Sun Yat-Sen Memorial Hospital, Sun Yat-Sen University, Guangzhou 510120, China; 3 Department of Drug Clinical Trial Institution, National Cancer Center/National Clinical Research Center for Cancer/Cancer Hospital & Shenzhen Hospital, Chinese Academy of Medical Sciences and Peking Union Medical College, Shenzhen 518116, China; 4 Department of Obstetrics and Gynecology, Sun Yat-Sen Memorial Hospital, Sun Yat-Sen University, Guangzhou 510120, China; 5 Bioscience and Biomedical Engineering Thrust, The Hong Kong University of Science and Technology (Guangzhou), Nansha, Guangzhou 511400, Guangdong, China; 6 Guangdong Provincial Clinical Research Center for Urological Diseases, Sun Yat-Sen Memorial Hospital, Sun Yat-Sen University, Guangzhou 510120, China; 7 Department of Urology, The Sixth Affiliated Hospital of Guangzhou Medical University, Qingyuan People's Hospital, Qingyuan 511518, Guangdong, China

## Abstract

Prostate cancer (PCa) is the second most common malignant tumor among males. Traditional treatments for PCa, which include surgery and endocrine therapy, have shown limited success, and more effective therapies are needed. Cisplatin (DDP) is an approved chemotherapeutic drug that causes DNA damage in cancer, whereas AZD7762, an inhibitor of CHK1, can significantly inhibit DNA repair. The effective therapeutic combination of cisplatin and the DNA damage response inhibitor AZD7762 has been considered to be a potential solution to the resistance to cisplatin and the adverse reactions that occur in many cancers. However, the co-transmission of cisplatin and AZD7762 and the unsatisfactory tumor-targeting efficacy of this therapy remain problems to be solved. Here, we confirmed the combined therapeutic efficacy of cisplatin and AZD7762 in PCa. Furthermore, we show that the glutathione-targeted Cys8E nanoparticles we synthesized, which have high drug-loading capacity, remarkable stability, and satisfactory release efficiency, enhanced the therapeutic efficacy of this treatment and reduced the required dosages of these drugs both *in vitro* and *in vivo*. Overall, we propose combination therapy of cisplatin and AZD7762 for PCa and facilitate it using Cys8E nanoparticles, which allow for better drug loading release, higher release efficiency, and more accurate tumor-targeting efficacy.

## I. INTRODUCTION

Prostate cancer (PCa) is the second most common cancer diagnosed among males, causing almost 10% of deaths in men diagnosed with cancer.^1^ Currently, the main treatment for PCa is androgen-deprivation therapy (ADT), which shows a promising curative effect in the short term.^1,2^ However, after two to three years of ADT, most cases progress to castration-resistant prostate cancer (CRPC), for which there is a lack of effective treatments.^3,4^ Cisplatin (DDP)-based chemotherapy has a significant antitumor effect and is widely available for the treatment of various malignant tumors, including testicular cancer, bladder cancer, lung cancer, and cervical cancer.^5–7^ However, DDP is not the main therapy for PCa, as PCa is insensitive to DDP, and the adverse effects of high-dose DDP are nonnegligible.

The molecular mechanism of the antitumor effect of DDP mainly involves DNA damage.^8,9^ DDP binds to purine residues of DNA, causing DNA damage, which further leads to apoptotic cell death.^10^ However, the DNA damage response (DDR) plays a key regulatory role in PCa.^11^ Our previous study showed that the TopBp1, a mediator of DNA damage repair, was highly expressed in PCa and modulated the progression of PCa via the ATR–CHK1 signaling pathway.^12^ This may partly explain why PCa is insensitive to DDP. It also demonstrates that inhibiting the DNA damage repair is a potential solution to achieve the application of DDP chemotherapy in PCa. Moreover, AZD7762, an inhibitor of CHK1, has been proven to enhance DDP cytotoxicity and to overcome DDP resistance in small cell lung cancer, osteosarcoma, and other malignant tumors.^13,14^ All these results indicate the potential anti-PCa effect of the combination of AZD7762 and DDP. Although the combination of these two drugs resulted in an improved anti-tumor effect in many malignant tumors, high doses of DDP and AZD7762 were required. Therefore, a co-delivery system for DDP and AZD7762 that could enhance the stability and tumor-targeting efficacy of the two drugs is essential for successful clinical applications of this combination in PCa treatment.

Nanoparticle (NP)-based drug delivery systems are extensively used in the treatment of cancer owing to their various advantages, which include their ability to target cancer cells by binding to the biomarkers, protecting drugs from degradation, and enabling reductions in both dose and toxicity.^15,16^ Owing to the higher glutathione (GSH) levels in many tumor tissues, including PCa, GSH-targeted NPs are extensively used as drug delivery systems for tumor therapy, resulting in significant curative effects.^17,18^

**Scheme 1.**
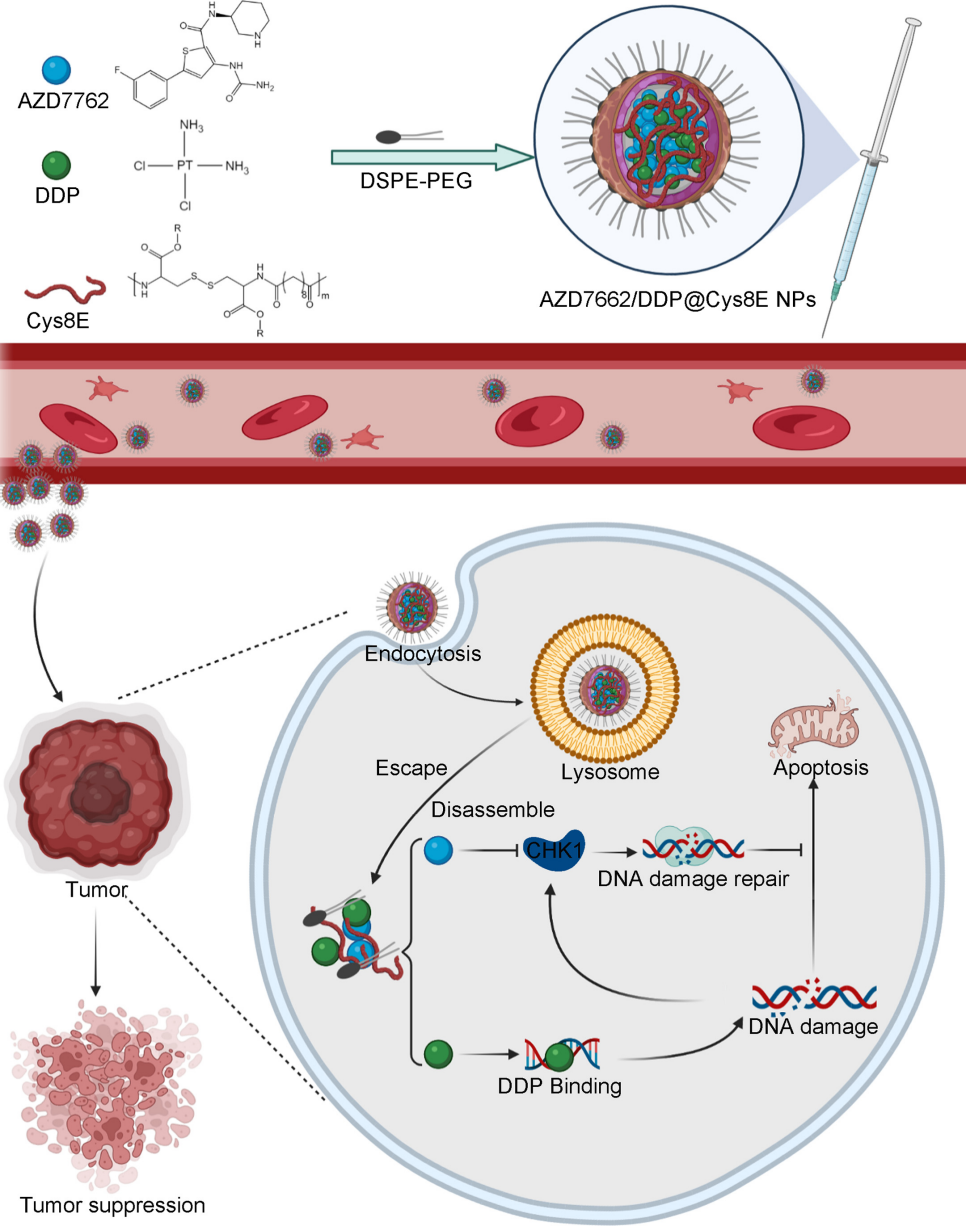
Scheme of the synthesis of AZD7662/DDP@Cys8E NPs (AD@Cys-8E NPs) and their therapeutic mechanism *in vivo*. Targeting the overexpressed GSH in PCa, AD@Cys-8E NPs accumulated in PCa and then rapidly disassembled and released AZD7762 and DDP. DDP causes DNA damage by binding to DNA, whereas AZD7762 inhibits DNA damage repair by inhibiting CHK1; therefore, the combination of AZD7762 and DDP enhances the therapeutic effect.

In this study, we synthesized a new hydrophobic poly polymer (Cys8E) as a GSH-targeted nanoplatform to carry AZD7762 and DDP. The Cys8E NPs showed high drug loading capacity, remarkable stability, and satisfactory release efficiency. We found that the combination of AZD7762 and DDP presented a more satisfactory curative effect in PCa than the application of AZD7762 or DDP independently. Moreover, the Cys8E NPs significantly increased the accumulation of AZD7762 and DDP in PCa, resulting in a more significant antitumor effect both *in vitro* and *in vivo* (Scheme 1).

## II. RESULTS

### A. Characterization of Cys8E NPs

**FIG. 1.**
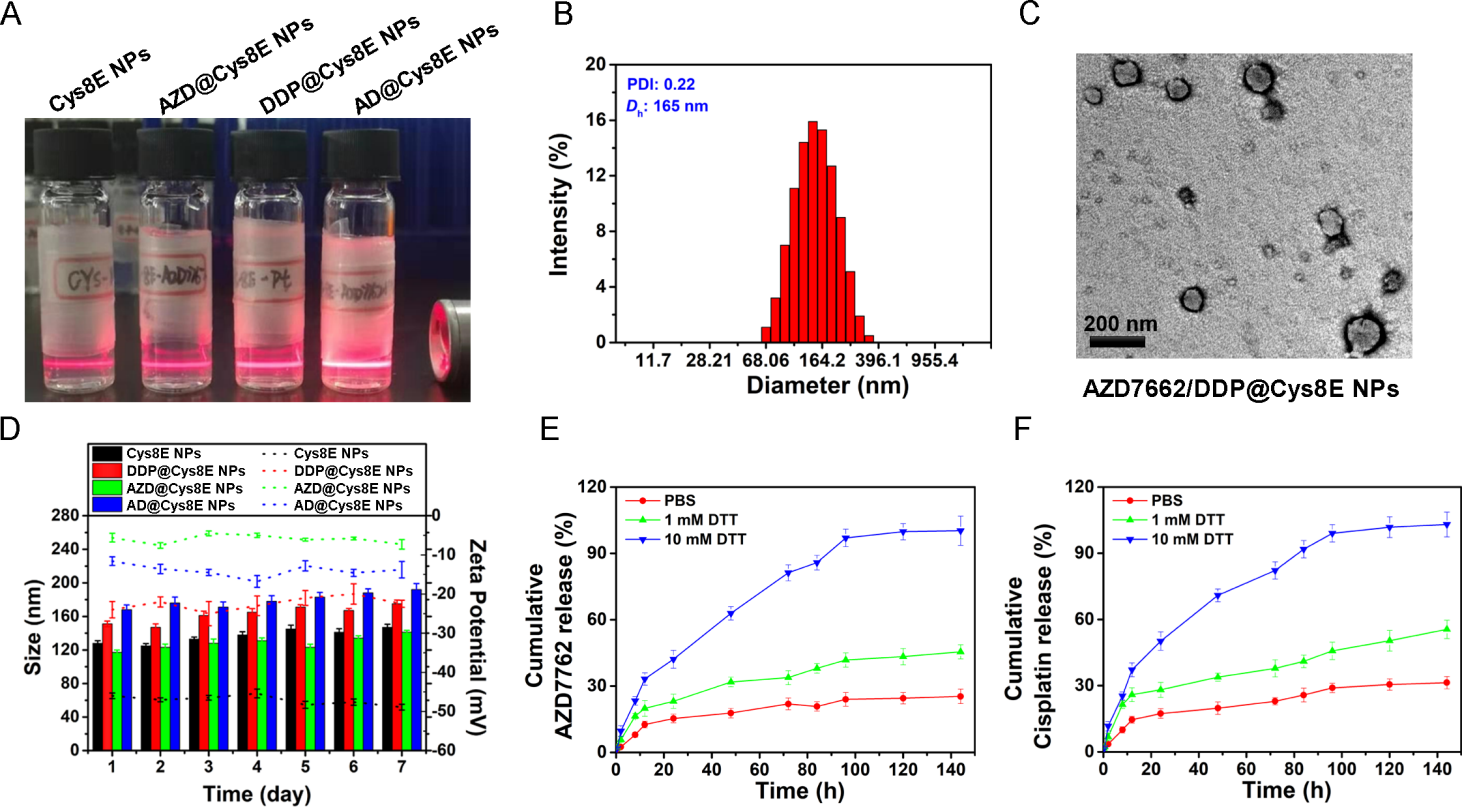
Characterization of AZD7662/DDP@Cys8E NPs. (a) Macroscopic characteristics of Cys8E NPs. (b) Size distribution of AD@Cys-8E NPs. (c) Representative transmission electron microscopy images of AD@Cys-8E NPs. (d) Stability of Cys8E NPs and related NPs in complete medium (RPMI 1640% + 10% FBS). (e) and (f) Cumulative release curve of AZD7662 and DDP in AD@Cys-8E NPs.

Cys8E, a derivative of the poly(disulfide amine) family, has been used as a drug carrier for the treatment of cancers and showed excellent biocompatibility and redox sensitivity. As expected, the drug@Cys8E NPs [Fig. 1(a) and supplementary material Fig. 1] showed satisfactory loading capacity and encapsulation efficiency, with AZD7762 loading capacities of 9.6% ± 1.5% and 10.7% ± 2.3% in AZD7662@Cys8E NPs (AZD@Cys8E NPs) and AZD7662/DDP@Cys8E NPs (AD@Cys8E NPs), respectively, and DDP loading capabilities of 21.3% ± 1.8% and 11.5% ± 2.2% in DDP@Cys8E NPs and AD@Cys8E NPs, respectively (Table I). Moreover, transmission electron microscopy images showed no significant changes in spherical morphology or minute changes in particle size in drug-Cys8E NPs compared with blank Cys8E NPs [Table II, Figs. 1(b) and 1(c)]. Further assessment showed that the particle size and zeta potential of Cys8E NPs with and without drugs remained essentially unchanged over 7 days [Fig. 1(d)].

**TABLE I.** Loading capacity and encapsulation efficiency of Cys-8E NPs.

Sample	Concentration of AZD7762 (*μ*g/ml)	Loading capacity (LC) of AZD7762 (%)	Encapsulation efficiency (EE) of AZD7762 (%)	Concentration of Pt (*μ*g/ml)	LC of Pt (%)	EE of Pt (%)
DDP@Cys8E NPs	⋯	⋯	⋯	75.2 ± 5.6	21.3 ± 1.8	7.1 ± 2.0
AZD@Cys8E NPs	155.0 ± 15.6	9.6 ± 1.5	28.7 ± 1.7	⋯	⋯	
AD@Cys8E NPs	159.0 ± 10.3	10.7 ± 2.3	34.8 ± 1.9	70.1 ± 3.8	11.5 ± 2.2	6.2 ± 1.3

**TABLE II:** Particle size of Cys-8E NPs.

Sample	Polymer dispersity index (PDI)	Mean intensity (d nm)	Mean count rate
Blank	0.23 ± 0.03	147.8 ± 12.3	109.0 ± 7.5
DDP @Cys8E NPs	0.15 ± 0.03	129.0 ± 10.5	112.1 ± 8.3
AZD@Cys8E NPs	0.16 ± 0.02	130.7 ± 11.7	65.7 ± 5.6
AD@Cys8E NPs	0.17 ± 0.02	164.6 ± 8.9	221.4 ± 4.8

As the high loading capacity and stability of Cys8E NPs had been demonstrated, we subsequently explored the release efficiency of Cys8E NPs as a drug carriers. As a GSH-targeted nanoplatform, Cys8E NPs were sensitive to the redox environment in tumors; thus, we used Dithiothreitol (DTT) to simulate the redox environment to assess the drug release profile of Cys8E NPs. The NPs were able to rapidly release much more AZD7762 or DDP in the presence of a high concentration of DTT [Figs. 1(e) and 1(f)]. These results demonstrate that Cys8E NPs represent a potential transport medium for AZD7662 and DDP owing to their high drug loading capacity, remarkable stability, and satisfactory release efficiency.

### B. High uptake kinetics of Cys8E NPs

**FIG. 2.**
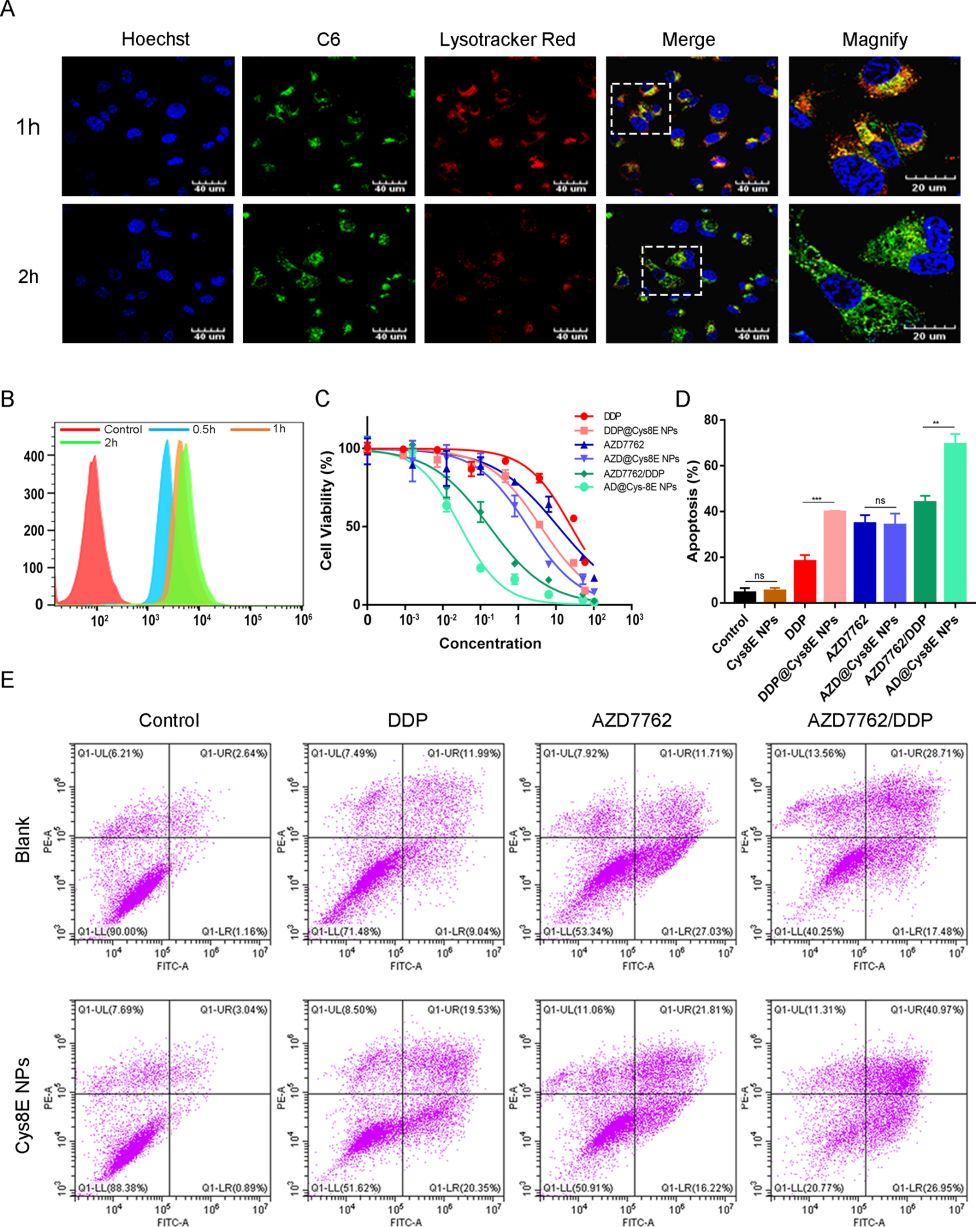
Cys8E NPs enhanced the therapeutic efficacy of AZD7662 and DDP. (a) Representative image of the distribution of Cys8E NPs in PC3 cells. (b) The cellular uptake analysis of PC3 cells by flow cytometry after incubation with Cys8E NPs. (c) PC3 cell viability after incubation with free drug or drug@Cys8E NPs for 24 h. (d) and (e) The apoptosis of PC3 cells *in vitro* was analyzed by flow cytometry.

For further investigation of the drug release profile of Cys8E NPs in PCa cells, we used a LysoTracker Red probe and fluorescent dye C6 to mark the lysosomes and NPs, respectively. After incubation for 1 h, significant green fluorescence, representing C6-marked Cys8E NPs, was observed in PC3 cells, indicating that Cys8E NPs were taken up quickly by PCa cells. We also observed remarkable colocalization of Cys8E NPs and lysosomes, which suggested that Cys8E NPs were taken up by cells by endocytosis. However, this raised a concern as to whether or not the lysosomes would disintegrate the NPs and degrade the carried drugs. Therefore, continuous observation was conducted. The significant green fluorescence persisted in the cytoplasm, and no further colocalization of Cys8E NPs and lysosomes was observed after incubation for 2 h. These observations confirmed that Cys8E NPs were able to escape from the lysosomes, which protected them from degradation and elimination [Fig. 2(a)]. These results showed that drugs carried by Cys8E NPs could be taken up by PCa cells efficiently and maintain a high concentration.

Cytometry analysis further confirmed this conclusion. Significant fluorescence intensity of Cys8E NPs was detected after incubation for 30 min and remained at a high level after 2 h [Fig. 2(b)], suggesting remarkable uptake kinetics of Cys8E NPs.

### C. Enhanced therapeutic efficacy caused by Cys8E NPs and the combination of AZD7762 and DDP *in vitro*

We next further explored the therapeutic efficacy of the combination of AZD7762 and DDP with or without Cys8E NPs as a delivery system. As expected, the AZD7662/DDP group (half-maximal inhibitory concentration (IC50): 0.17 *μ*M AZD7662 and 0.10 *μ*M DDP) indicated much lower cell viability compared with the AZD766 group (IC50: 11.39 *μ*M AZD7662) and the DDP group (IC50: 25.25 *μ*M DDP). This demonstrated that the combination of AZD7762 and DDP was a much better treatment than AZD7762 or DDP administered alone. Moreover, Cys8E NPs further improved the therapeutic efficacy of the combination or single use of AZD7762 and DDP [Fig. 2(c)].

Then, apoptosis assays were conducted to further explore the therapeutic efficacy of Cys8E NPs. As shown in Figs. 2(d) and 2(e), more apoptosis was found in the AZD7662/DDP group than in the AZD766 group or DDP group. Unsurprisingly, Cys8E NPs increased the apoptosis of PCa cells when used as a drug delivery system, whereas Cys8E NPs without drugs had little impact on PCa cells. Notably, there was no difference between the total apoptosis rate of the AZD7662 group and that of the AZD@Cys8E NPs group. However, the apoptosis rate at the late stage was significantly increased in the AZD@Cys8E NPs group, indicating that AZD@Cys8E NPs caused significant apoptosis, whereas Cys8E NPs had little impact on PCa cells. All these results demonstrate that it was the delivery capacity of Cys8E NPs that enhanced the therapeutic efficacy of AZD7762 and DDP, rather than the toxicity of NPs.

### D. Targeting function of Cys8E NPs

**FIG. 3.**
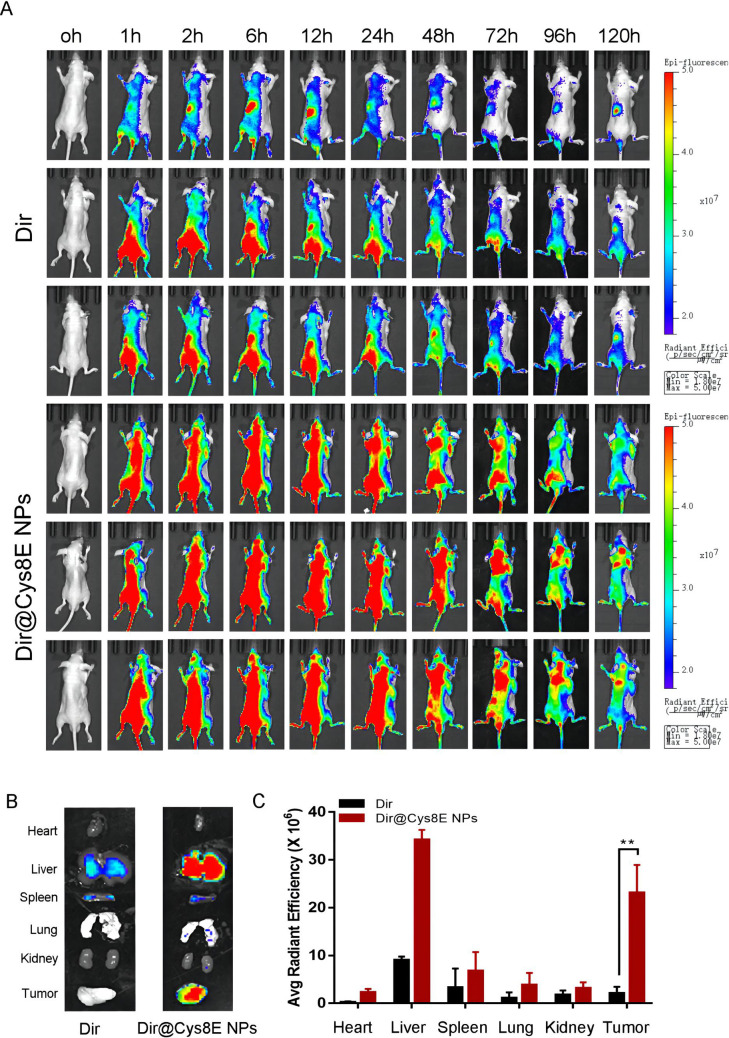
Biodistribution of Cys8E NPs *in vivo*. (a) Representative image of the distribution of free Dir and Dir@Cys8E NPs in PC3-tumor-bearing mice. (b) Fluorescence images of major organs and tumors extracted from PC3-tumor-bearing mice at 120 h. (c) Quantitative analysis of the fluorescent signals from the major organs and tumors from PC3-tumor-bearing mice treated with free Dir and Dir@Cys8E NPs.

To determine the targeting function of Cys8E NPs in PCa, PC3-tumor-bearing mice were treated with free Dir or Dir@Cys8E NPs through tail vein injection. Then, the distribution and density of Dir were detected by an IVIS Imaging System at different time points. As shown in Fig. 3(a), free Dir mostly accumulated in the liver. Although a weak fluorescence signal was detected in tumor tissue in some mice injected with free Dir, this soon became undetectable. By contrast, significantly increased Dir levels were detected in both liver and PCa tissue in mice injected with Dir carried by Cys8E NPs, and these remained high for several days.

To observe the distribution of Dir in different organs more directly and reduce the impact of soft tissue on observation, the main organs and tumor tissues were isolated and imaged for further quantitative evaluation. Consistent with the results described above, the fluorescence signal of Dir carried by Cys8E NPs was extremely enhanced in PCa tissue and remained at a high level for several days, whereas free Dir was not detected in PCa tissue [Figs. 3(b) and 3(c)]. All these results indicated a remarkable targeting function of Cys8E NPs in PCa, suggesting that Cys8E NPs is a promising drug delivery systemin PCa. The fluorescence signal of Dir in the liver was also enhanced by Cys8E NPs, possibly owing to Kupffer cells and the strong redox reaction in the liver, and this raised a concern as to whether Cys8E NPs would have an impact on the liver during treatment. However, further experiments showed that Cys8E NPs did not cause extra damage to the liver, possibly owing to the strong detoxification ability of the liver.

### E. Enhanced therapeutic efficacy *in vivo*

**FIG. 4.**
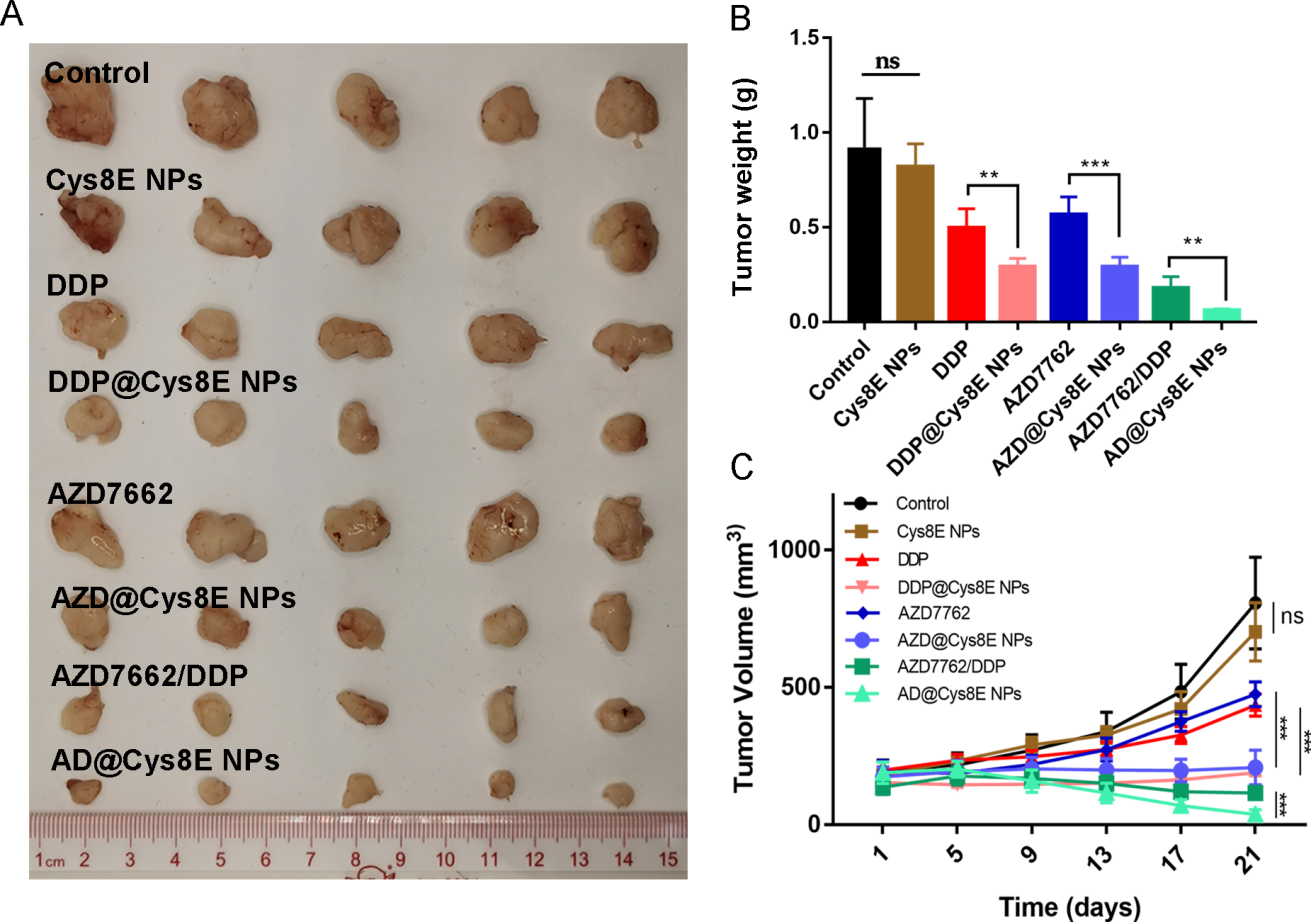
Cys8E NPs enhanced the antitumor efficacy of AZD7662 and DDP *in vivo*. (a) Magnification of tumors excised from mice treated with PBS, Cys8E NPs, DDP, DDP @Cys8E NPs, AZD7662, AZD@Cys8E NPs, AZD7662/DDP, or AD@Cys8E NPs for 21 days. (b) Weights of tumors in each group. (c) Tumor volumes during the treatments.

**FIG. 5.**
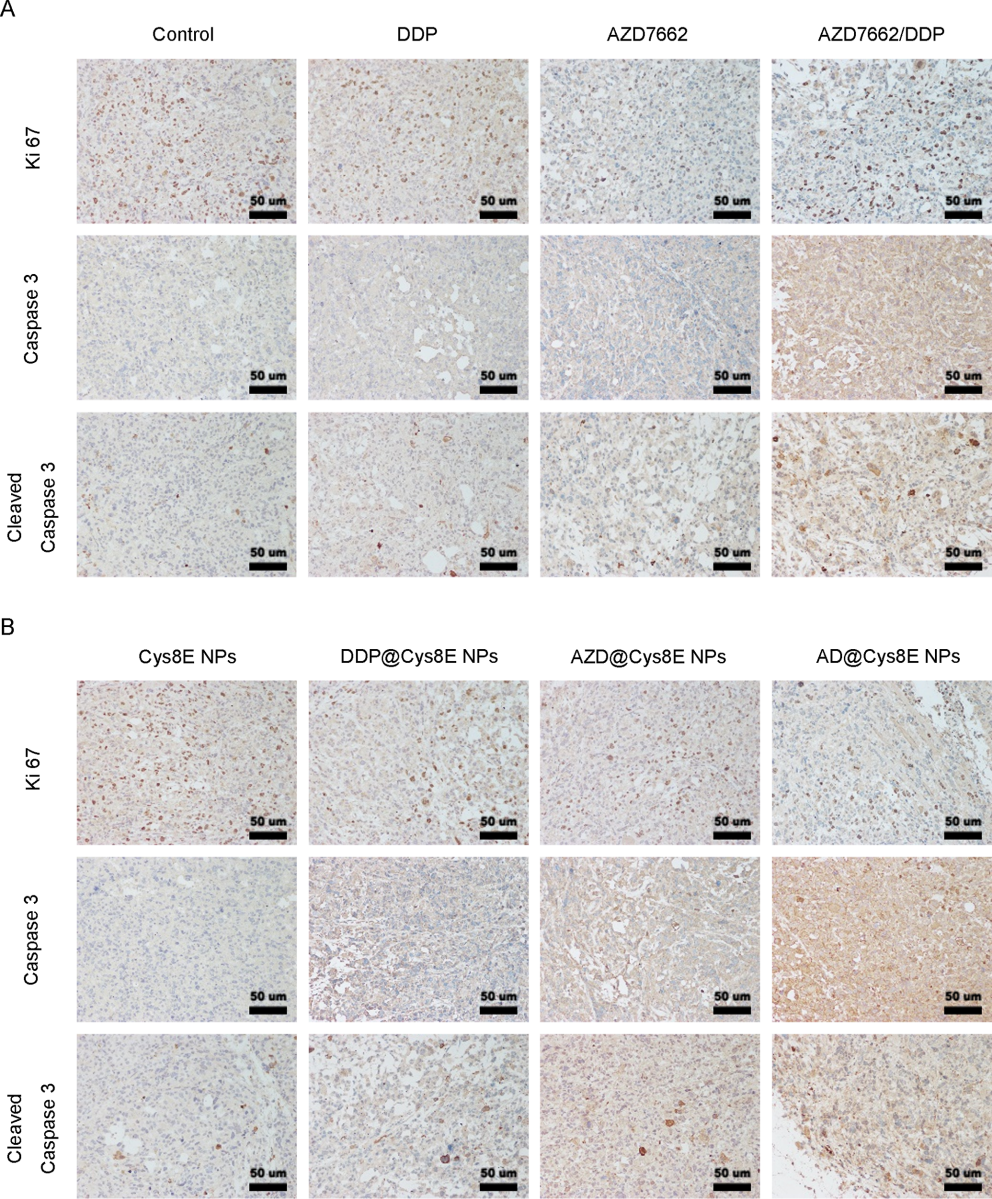
Cys8E NPs enhanced the antitumor efficacy of AZD7662 and DDP *in* PCa. IHC examination of Ki67, caspase 3, and cleaved caspase 3 expressed in tumors from mice treated with PBS, DDP, AZD7662, AZD7662/DDP (a), Cys8E NPs, DDP @Cys8E NPs, AZD@Cys8E NPs, or AD@Cys8E NPs (b). Scale bar: 50 *μ*m.

Having demonstrated the enhanced therapeutic efficacy of Cys8E NPs and the combination of AZD7762 and DDP *in vitro*, we next verified their therapeutic efficacy *in vivo*. PC3-tumor-bearing mice were injected with phosphate-buffered saline (PBS), Cys8E NPs, DDP, DDP@Cys8E NPs, AZD7662, AZD@Cys8E NPs, AZD7662/DDP, or AD@Cys8E NPs every 2 days by intravenous injection. The results showed that AZD7662/DDP significantly reduced tumor volume and weight, and that drug-NPs had much better therapeutic efficacy than drugs without Cys8E NPs [Figs. 4(a)–4(c)]. Tumors were further analyzed by IHC, and similar results were found: treatment with Cys8E NPs and AZD7662/DDP reduced the expression of Ki67 in tumors while increasing the expression of caspase-3 and cleaved caspase3 [Figs. 5(a) and 5(b)].

**FIG. 6.**
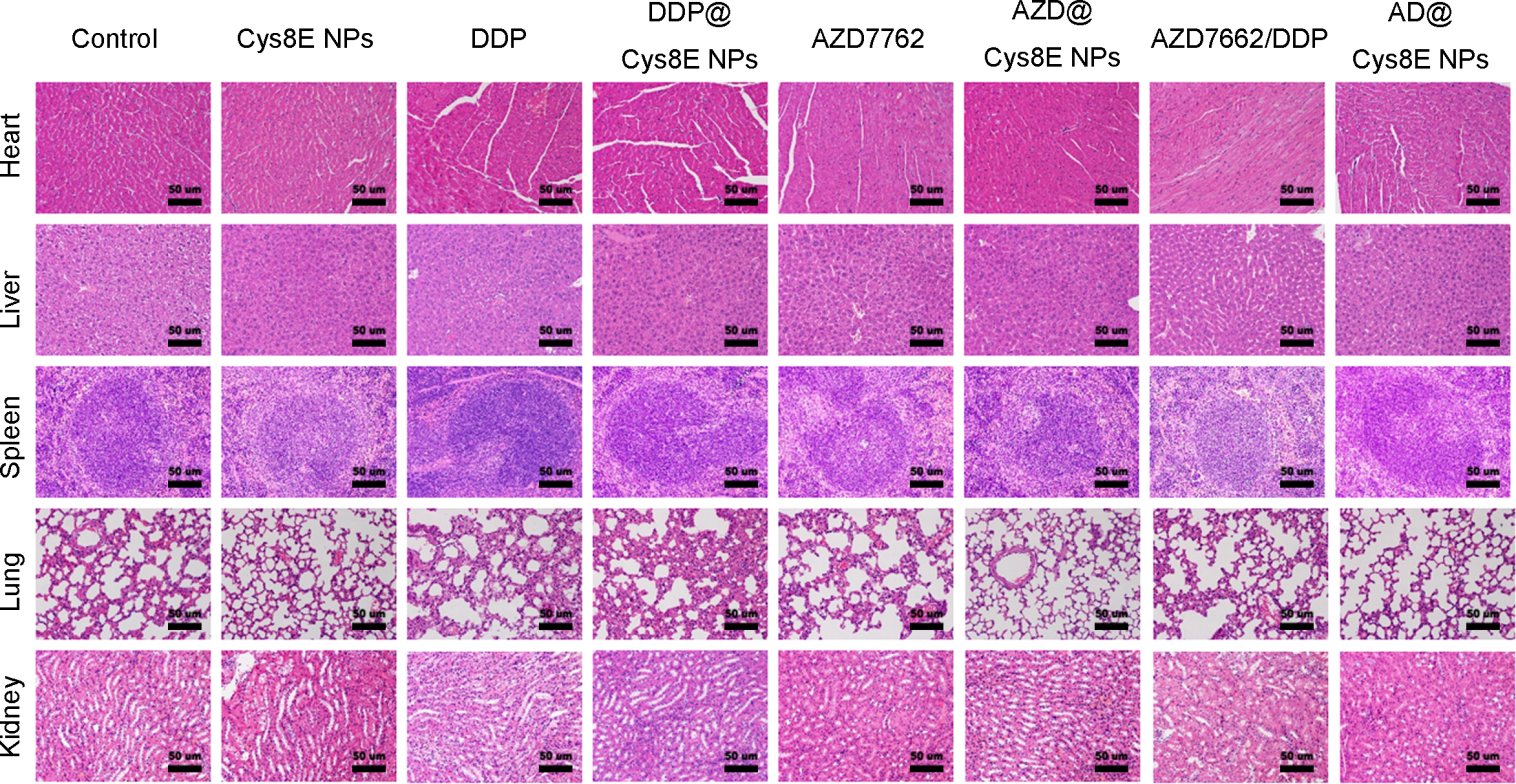
Cys8E NPs did not cause extra organ damage. H&E staining of major organs of PC3-tumor-bearing mice in each group. Scale bar: 50 *μ*m.

As discussed above, the adverse effects of DDP are nonnegligible; therefore, we sought to verify whether the use of Cys8E NPs could reduce or enhance these adverse effects. As shown in Fig. 6, hematoxylin and eosin (H&E) staining showed no significant changes in major organs among the eight groups. In addition, further analysis of the serum of PC3-tumor-bearing mice showed that alanine aminotransferase (ALT), aspartate aminotransferase (AST), blood urea nitrogen (BUN), and creatinine (CREA) levels were all within normal ranges [supplementary material Figs. 2(a) and 2(b)]. Although DDP and the combination of AZD7762 and DDP caused slightly elevated AST and BUN, but not beyond the normal range, the application of Cys8E NPs did not have further impact on major organs. These results confirmed that Cys8E NPs could enhance the therapeutic efficacy of DDP and AZD7762 with favorable biosafety.

## III. DISCUSSION

PCa is an urgent challenge among male cancers, with an incidence rate that continues to rise. The major treatment for PCa is endocrine therapy; however, a large number of PCa patients progress to CRPC, for which treatment is limited. Therefore, new treatments for PCa are urgently required.^22–24^ The DDR is involved in all stages of PCa, and targeting the DDR is a new but promising approach to PCa treatment.^25–27^ DDP, a widely used chemotherapeutic drug, is one of the most commonly used DNA-damaging agents for cancer treatment. However, the use of DDP in PCa has been limited by its poor therapeutic efficacy and the adverse reactions. The strong DDR in PCa may be the reason for the poor therapeutic efficacy of DDP, based on the mechanisms of the antitumor mechanism of DDP and of resistance to this drug.^28–30^ Therefore, inhibition of the DDR is a potential approach to improve the treatment of PCa. AZD7762 is an inhibitor of CHK1, which has been proven to be a DDR regulator and an important regulator in PCa.^12,31,32^ Herein, we report that the combination of DDP and AZD7762 could markedly inhibit the progression of PCa, consistent with previous reports on many cancers.^13,14,33,34^ Despite the remarkable therapeutic efficacy, there were some limitations to the application of the combination of DDP and AZD7762. For example, the dose needed to be decreased, and a more targetable delivery system was required.

Traditional drugs for cancer treatment face various challenges, including degradation in circulation, nonspecific enrichment, and poor targeting effects. Therefore, many researchers have explored the potential of new and effective drug delivery systems.^35–37^ Nanomaterials are among the most promising solutions to this problem. With the protection of nanometer materials, loaded drugs can remain nondegradable. Nanometer materials can also disassemble efficiently in the specific tumor environment, enabling high enrichment of the drug in the tumor. Notably, nanometer materials can be modified for combination with tumor markers to significantly enhance the targeting effect.^38–40^ Recently, GSH-targeted NPs have been widely used in the delivery of antitumor drugs. On the one hand, the GSH-targeted NPs could target to GSH, which is elevated in many cancers and is among the causes of chemotherapy resistance; on the other hand, the superiority of GSH-targeted NPs was demonstrated with respect to various aspects, including greater drug loading, faster drug release, and improved safety.^41,42^ In this study, we synthesized a new hydrophobic poly polymer (Cys8E) as a GSH-targeted nanoplatform. The resulting Cys8E NPs showed high drug loading capacity, satisfactory release efficiency, and remarkable targeting function, owing to their particle size and high sensitivity to GSH.

As the delivery efficiency of Cys8E NPs was proved to be satisfactory, the other concern about this nano platform was the biosafety of Cys8E NPs. As we observed from the apoptosis assays, Cys8E NPs did not cause more apoptosis in PCa cells than the negative control, which means Cys8E NPs was harmless to cells. Also, the hematoxylin-eosin staining (HE staining) of major organs showed no significant edema, atrophy, and necrosis in the cells among the mice treated with blank Cys8E NPs. On the other hand, blank Cys8E NPs did not cause the rise of ALT, AST, BUN, and CREA, compared with the negative control. Therefore, Cys8E NPs was both efficient and safe, which was able to facilitate the clinical applications of the combination of DDP and AZD7762 in PCa.

## IV. CONCLUSIONS

In summary, the AZD7662/DDP-loaded Cys8E NPs we synthesized as a GSH-targeted nanoplatform were based on hydrophobic poly polymers. AZD7662/DDP-loaded Cys8E NPs showed high drug loading, remarkable stability, and satisfactory release efficiency as well as a remarkable targeting function. They also displayed great therapeutic efficacy against PCa in both cell lines and xenografts. Therefore, AZD7662/DDP@ Cys8E NPs have potential as an effective therapy for PCa.

## V. METHODS

### A. Cell line and cell culture

PCa cell line PC3 was purchased from Cell Bank/Stem Cell Bank, Chinese Academy of Sciences. PC3 cells were cultured in RPMI-1640 medium (Gibco, Shanghai, China) with 10% fetal bovine serum (FBS) and penicillin/streptomycin (MCE, Shanghai, China) in a humidified incubator at 37 °C with 5% CO_2_.

### B. Chemicals and reagents

AZD7762 (S1532) was purchased from Selleck (Shanghai, China); DDP was purchased from MCE (Shanghai, China). This study used antibodies against caspase-3 (Servicebio, Wuhan, China), cleaved caspase-3 (Servicebio, Wuhan, China), and Ki67 (Servicebio, Wuhan, China).

### C. Synthesis and analysis of NPs

DDP@Cys8E NPs, AZD7662@Cys8E NPs, AZD7662/DDP@Cys8E NPs, and blank Cys8E NPs were synthesized by a nanoprecipitation method. First, Cys8E solutions in dimethyl sulfoxide (DMSO), 1,2-distearoyl-sn-glycero-3-phosphoethanolamine-N-[biotinyl(polyethylene glycol)-2000] (DSPE-PEG 2000), AZD7762, and DDP were mixed; then, the mixture was blended with ultrapure water by stirring. After centrifugation twice, the NPs were collected. Then, the parameters of NPs, including particle size, zeta potential, and morphology, were further measured by dynamic light scattering (Zetasizer Nano-ZS90, Malvern) and transition electron microscopy (JEM-1400 Plus, JEOL). The encapsulation efficiency and loading capacity of NPs were calculated by the following formulas as described previously:^19^

$$\begin{array}{c} \mathrm{Encapsulation}\;\mathrm{efficiency}\;\left(\%\right)=\frac{\mathrm{loaded}\frac{\mathrm{AZD}7662}{\mathrm{DDP}}}{\;\mathrm{feeding}\frac{\mathrm{AZD}7662}{\mathrm{DDP}}},\\ \mathrm{Loading}\;\mathrm{capacity}\left(\%\right)=\frac{\mathrm{loaded}\frac{\mathrm{AZD}7662}{\mathrm{DDP}}}{\mathrm{feeding}\;\mathrm{NPs}}. \end{array}$$

### D. *In vitro* release profiles

The drug-release behavior of NPs was detected as previously described.^20^ In brief, NPs were transferred into a dialysis tube and stimulated by different concentrations of DTT at 37 °C. Then, high-performance liquid chromatography was used to analyze the amounts of drug released.

### E. Cellular uptake assay

PC3 cells were cultured in a six-well plate (1 × 10^5^ cells per well) for 2 days. Then, C6-loaded Cys8E NPs were added to the plates. After incubation for 2 h, the efficiency of cellular uptake was detected by flow cytometry.

### F. Distribution of Cys8E NPs in PC3 cells

PC3 cells were seeded in a six-well plate (1 × 10^5^ cells per well) and cultured overnight. Next, the medium was replaced with C6-loaded NPs in serum-free medium. After incubation for 1 or 4 h, the cells were washed twice with phosphate-buffered saline (PBS) and then treated with Hoechst 33342 (5 *μ*g/ml) and LysoTracker Red (75 nM) sequentially. Finally, the cells were visualized by confocal laser scanning microscope.

### G. *In vitro* cytotoxicity stud*y*

PC3 cells were seeded in 96-well plates at a density of 3000 cells per well. Twenty-four hours later, the cells were treated with AZD7662, DDP, AZD7662/DDP, DDP@Cys8E NPs, AZD7662@Cys8E NPs, or AZD7662/DDP@Cys-E NPs at different concentrations. After 48 h of incubation, the medium was removed, and 10 *μ*l of CCK8 in 100 *μ*l of RPMI-1640 medium was added to each well. After 2 h of incubation, the optical densities at 450 nm were determined using a microplate reader (Multiskan MK3; Thermo Scientific, Shanghai, China).

### H. Apoptosis assay

PC3 cells were seeded in six-well plates at a density of 200 000 cells per well and cultured for 24 h. The cells were next treated with PBS, AZD7662 (6.4 *μ*M), DDP (3.6 *μ*M), AZD7662/DDP (AZD7662: 6.4 *μ*M, DDP: 3.6 *μ*M), Cys8E NPs, AZD7662@Cys8E NPs (AZD7662: 6.4 *μ*M), DDP@Cys8E NPs (DDP: 3.6 *μ*M), or AZD7662/DDP@Cys8E NPs (AZD7662: 6.4 *μ*M, DDP: 3.6 *μ*M) for 48 h. Then, the cells were collected, and apoptosis was detected using an Annexin V/FITC apoptosis detection kit.^12^

### I. *In vivo* biodistribution study

PC3-tumor-bearing mice were injected with Dir or Dir@Cys8E NPs through the caudal vein. The distribution and density of Dir were detected using an IVIS Imaging System at different time points. Finally, mice were dissected, and the major organs and tumors were obtained to further detect the distribution and density of Dir at 120 h after injection.

### J. Tumor-bearing mouse model and treatments

All experiments involving mice were approved by the Institutional Animal Care and Use Committee of Sun Yat-Sen University. BALB/c nude mice (male, four to five weeks old) were purchased from Charles River (Beijing, China). Mice were divided into eight groups, with five mice per group. PC3 cells (2 × 10^6^) were injected subcutaneously into the right side of the dorsum.^21^ When the tumor volume reached 50 mm^3^, the following drugs were injected into mice through the tail vein with a dose of 20 mg AZD7662/kg or 5 mg DDP/kg every two days: (i) PBS, (ii) Cys8E NPs, (iii) DDP, (iv) DDP@Cys8E NPs, (v) AZD7662, (vi) AZD7662@Cys8E NPs, (vii) AZD7662/DDP, and (viii) AZD7662/DDP@Cys8E NPs.

### K. Immunohistochemistry

Expression levels of caspase-3, cleaved caspase-3, and Ki67 were detected to assess the proliferation and apoptosis of tumor tissues following standard procedures using immunohistochemistry (IHC). IHC images were taken with a Nikon Eclipse Ni-U system and NIS Elements software (Nikon, Tokyo, Japan).

### L. Statistical analyses

Quantitative data are presented as means ± SD. One-way analysis of variance and unpaired Student's *t-*test were used for comparisons between groups. GraphPad Prism 8.0 (GraphPad, La Jolla, CA, USA) was used to perform all the statistical analyses in this study. *P* <0.05 was considered to indicate statistical significance: ^*^P <0.05, ^**^P <0.01, and ^***^P <0.001.

## SUPPLEMENTARY MATERIAL

See the supplementary material for the synthesis of Cys-8E polymer, the ^1^H NMR results of Cys8E, and the serum analysis of PC3 tumor-bearing mice.

## Data Availability

The data that support the findings of this study are available within the article and its supplementary material.

## References

[1] E. D. Crawford , A. Heidenreich , N. Lawrentschuk , B. Tombal , A. C. L. Pompeo , A. Mendoza-Valdes , K. Miller , F. M. J. Debruyne , and L. Klotz , Prostate Cancer Prostatic Dis. 22, 24–38 (2019). 10.1038/s41391-018-0079-0 30131604 PMC6370592

[2] A. J. Evans , Mod. Pathol. 31, S110–S121 (2018). 10.1038/modpathol.2017.158 29297495

[3] M. Y. Teo , D. E. Rathkopf , and P. Kantoff , Annu. Rev. Med. 70, 479–499 (2019). 10.1146/annurev-med-051517-011947 30691365 PMC6441973

[4] A. A. Shafi , A. E. Yen , and N. L. Weigel , Pharmacol. Ther. 140, 223–238 (2013). 10.1016/j.pharmthera.2013.07.003 23859952

[5] G. de Vries , X. Rosas-Plaza , M. van Vugt , J. A. Gietema , and S. de Jong , Cancer Treat. Rev. 88, 102054 (2020). 10.1016/j.ctrv.2020.102054 32593915

[6] S. Ghosh , Bioorg. Chem. 88, 102925 (2019). 10.1016/j.bioorg.2019.102925 31003078

[7] J. J. Coen , P. Zhang , P. J. Saylor , C. T. Lee , C. L. Wu , W. Parker , T. Lautenschlaeger , A. L. Zietman , J. A. Efstathiou , A. B. Jani , O. Kucuk , L. Souhami , J. P. Rodgers , H. M. Sandler , and W. U. Shipley , J. Clin. Oncol. 37, 44–51 (2019). 10.1200/JCO.18.00537 30433852 PMC6354769

[8] H. N. Fraval , C. J. Rawlings , and J. J. Roberts , Mutat. Res. 51, 121–132 (1978). 10.1016/0027-5107(78)90014-3 672924

[9] D. J. Beck and R. R. Brubaker , J. Bacteriol. 116, 1247–1252 (1973). 10.1128/jb.116.3.1247-1252.1973 4584807 PMC246480

[10] S. Dasari and P. B. Tchounwou , Eur. J. Pharmacol. 740, 364–378 (2014). 10.1016/j.ejphar.2014.07.025 25058905 PMC4146684

[11] J. Mateo , G. Boysen , C. E. Barbieri , H. E. Bryant , E. Castro , P. S. Nelson , D. Olmos , C. C. Pritchard , M. A. Rubin , and J. S. de Bono , Eur. Urol. 71, 417–425 (2017). 10.1016/j.eururo.2016.08.037 27590317

[12] K. Li , S. Peng , Z. Li , Y. Lai , Q. Wang , Y. Tao , W. Wu , Q. Zhou , Z. Gao , J. Chen , H. Li , W. Cai , Z. Guo , and H. Huang , Aging 12, 9948–9958 (2020). 10.18632/aging.103260 32459662 PMC7288942

[13] W. H. Hsu , X. Zhao , J. Zhu , I. K. Kim , G. Rao , J. McCutcheon , S. T. Hsu , B. Teicher , B. Kallakury , A. Dowlati , Y. W. Zhang , and G. Giaccone , J. Thorac. Oncol. 14, 1032–1045 (2019). 10.1016/j.jtho.2019.01.028 30771522 PMC6534433

[14] J. Zhu , H. Zou , W. Yu , Y. Huang , B. Liu , T. Li , C. Liang , and H. Tao , Cancer Cell Int. 19, 195 (2019). 10.1186/s12935-019-0896-9 31372095 PMC6660702

[15] J. Xie , Y. Lu , B. Yu , J. Wu , and J. Liu , Chin. Chem. Lett. 31, 1173–1177 (2020). 10.1016/j.cclet.2019.10.030

[16] Z. Li , W. Xu , J. Yang , J. Wang , J. Wang , G. Zhu , D. Li , J. Ding , and T. Sun , Adv. Mater. 34, e2200449 (2022). 10.1002/adma.202200449 35291052

[17] L. Wang , X. You , Q. Lou , S. He , J. Zhang , C. Dai , M. Zhao , M. Zhao , H. Hu , and J. Wu , Biomater. Sci. 7, 4218–4229 (2019). 10.1039/C9BM00907H 31389415

[18] M. Daga , C. Ullio , M. Argenziano , C. Dianzani , R. Cavalli , F. Trotta , C. Ferretti , G. P. Zara , C. L. Gigliotti , E. S. Ciamporcero , P. Pettazzoni , D. Corti , S. Pizzimenti , and G. Barrera , Free Radical Biol. Med. 97, 24–37 (2016). 10.1016/j.freeradbiomed.2016.05.009 27184956

[19] J. Q. Wang , L. Y. Wang , S. J. Li , T. Tong , L. Wang , C. S. Huang , Q. C. Xu , X. T. Huang , J. H. Li , J. Wu , W. Zhao , and X. Y. Yin , Nanoscale 12, 15767–15774 (2020). 10.1039/D0NR03138K 32729861

[20] Z. Li , J. Huang , T. Du , Y. Lai , K. Li , M. Luo , D. Zhu , J. Wu , and H. Huang , Chin. Chem. Lett. 33, 2496–2500 (2022). 10.1016/j.cclet.2021.11.078

[21] Z. Li , Q. Wang , S. Peng , K. Yao , J. Chen , Y. Tao , Z. Gao , F. Wang , H. Li , W. Cai , Y. Lai , K. Li , X. Chen , and H. Huang , Clin. Transl. Med. 10, e191 (2020). 10.1002/ctm2.191 33135357 PMC7536616

[22] F. Guo , C. Zhang , F. Wang , W. Zhang , X. Shi , Y. Zhu , Z. Fang , B. Yang , and Y. Sun , Cell Death Differ. 27, 1938–1951 (2020). 10.1038/s41418-019-0473-8 31857702 PMC7244590

[23] P. A. Watson , V. K. Arora , and C. L. Sawyers , Nat. Rev. Cancer 15, 701–711 (2015). 10.1038/nrc4016 26563462 PMC4771416

[24] T. A. Yap , A. Zivi , A. Omlin , and J. S. de Bono , Nat. Rev. Clin. Oncol. 8, 597–610 (2011). 10.1038/nrclinonc.2011.117 21826082

[25] A. M. Wengner , A. Scholz , and B. Haendler , Int. J. Mol. Sci. 21, 8273 (2020). 10.3390/ijms21218273 33158305 PMC7663807

[26] W. Abida , D. Campbell , A. Patnaik , J. D. Shapiro , B. Sautois , N. J. Vogelzang , E. G. Voog , A. H. Bryce , R. McDermott , F. Ricci , J. Rowe , J. Zhang , J. M. Piulats , K. Fizazi , A. S. Merseburger , C. S. Higano , L. E. Krieger , C. J. Ryan , F. Y. Feng , A. D. Simmons , A. Loehr , D. Despain , M. Dowson , F. Green , S. P. Watkins , T. Golsorkhi , and S. Chowdhury , Clin. Cancer Res. 26, 2487–2496 (2020). 10.1158/1078-0432.CCR-20-0394 32086346 PMC8435354

[27] W. Zhang , B. Liu , W. Wu , L. Li , B. M. Broom , S. P. Basourakos , D. Korentzelos , Y. Luan , J. Wang , G. Yang , S. Park , A. K. Azad , X. Cao , J. Kim , P. G. Corn , C. J. Logothetis , A. M. Aparicio , A. M. Chinnaiyan , N. Navone , P. Troncoso , and T. C. Thompson , Clin. Cancer Res. 24, 696–707 (2018). 10.1158/1078-0432.CCR-17-1872 29138344 PMC5823274

[28] C. Rudolph , C. Melau , J. E. Nielsen , K. Vile Jensen , D. Liu , J. Pena-Diaz , E. Rajpert-De Meyts , L. J. Rasmussen , and A. Jorgensen , Cell. Oncol. 40, 341–355 (2017). 10.1007/s13402-017-0326-8 PMC13001569 28536927

[29] B. Moolmuang and M. Ruchirawat , J. Pharm. Pharmacol. 73, 40–51 (2021). 10.1093/jpp/rgaa050 33791808

[30] M. Duan , J. Ulibarri , K. J. Liu , and P. Mao , Int. J. Mol. Sci. 21, 9248 (2020). 10.3390/ijms21239248 33291532 PMC7730652

[31] S. D. Zabludoff , C. Deng , M. R. Grondine , A. M. Sheehy , S. Ashwell , B. L. Caleb , S. Green , H. R. Haye , C. L. Horn , J. W. Janetka , D. Liu , E. Mouchet , S. Ready , J. L. Rosenthal , C. Queva , G. K. Schwartz , K. J. Taylor , A. N. Tse , G. E. Walker , and A. M. White , Mol. Cancer Ther. 7, 2955–2966 (2008). 10.1158/1535-7163.MCT-08-0492 18790776

[32] S. Karanika , T. Karantanos , L. Li , J. Wang , S. Park , G. Yang , X. Zuo , J. H. Song , S. N. Maity , G. C. Manyam , B. Broom , A. M. Aparicio , G. E. Gallick , P. Troncoso , P. G. Corn , N. Navone , W. Zhang , S. Li , and T. C. Thompson , Cell Rep. 18, 1970–1981 (2017). 10.1016/j.celrep.2017.01.072 28228262 PMC5349188

[33] Y. Meng , C. W. Chen , M. M. H. Yung , W. Sun , J. Sun , Z. Li , J. Li , Z. Li , W. Zhou , S. S. Liu , A. N. Y. Cheung , H. Y. S. Ngan , J. C. Braisted , Y. Kai , W. Peng , A. Tzatsos , Y. Li , Z. Dai , W. Zheng , D. W. Chan , and W. Zhu , Cancer Lett. 428, 104–116 (2018). 10.1016/j.canlet.2018.04.029 29704517 PMC7474466

[34] C. Y. Yang , C. R. Liu , I. Y. Chang , C. N. OuYang , C. H. Hsieh , Y. L. Huang , C. I. Wang , F. W. Jan , W. L. Wang , T. L. Tsai , H. Liu , C. P. Tseng , Y. S. Chang , C. C. Wu , and K. P. Chang , Cancers 12, 1726 (2020). 10.3390/cancers12071726 32610557 PMC7408003

[35] X. Y. Jun Huang , P. Xin , Z. Gu , C. Chen , and J. Wu , Chin. Chem. Lett. 32, 1737–1742 (2021). 10.1016/j.cclet.2020.12.006

[36] P. O. Ruixuan Chen , L. Su , X. Xu , P. Lian , Y. Li , Q. Gao , Y. Zhang , S. Nie , F. Luo , R. Xu , X. Zhang , X. Li , Y. Cao , P. Gao , J. Kang , J. Wu , and L. Li , Chin. Chem. Lett. 33, 4610–4616 (2022). 10.1016/j.cclet.2022.03.074

[37] G. L. X. Feng , W. Xu , X. Xu , J. Ding , and X. Chen , Sci. China Chem. 64, 293–301 (2020). 10.1007/s11426-020-9884-6

[38] C. Li , X. You , X. Xu , B. Wu , Y. Liu , T. Tong , J. Chen , Y. Li , C. Dai , Z. Ye , X. Tian , Y. Wei , Z. Hao , L. Jiang , J. Wu , and M. Zhao , Adv. Sci. 9, e2104134 (2022). 10.1002/advs.202104134 PMC8948613 35080145

[39] X. Xu , J. Wang , T. Tong , W. Zhang , J. Wang , W. Ma , S. Wang , D. Zhou , J. Wu , L. Jiang , and M. Zhao , Haematologica 107, 2344–2355 (2022). 10.3324/haematol.2021.280290 35295079 PMC9521229

[40] C. S. Huang , Q. C. Xu , C. Dai , L. Wang , Y. C. Tien , F. Li , Q. Su , X. T. Huang , J. Wu , W. Zhao , and X. Y. Yin , ACS Nano 15, 14744–14755 (2021). 10.1021/acsnano.1c04570 34405985

[41] L. Wang , X. Huang , X. You , T. Yi , B. Lu , J. Liu , G. Lu , M. Ma , C. Zou , J. Wu , and W. Zhao , Signal Transduction Targeted Ther. 5, 196 (2020). 10.1038/s41392-020-00248-x PMC7518281 32973147

[42] W. Wu , M. Chen , T. Luo , Y. Fan , J. Zhang , Y. Zhang , Q. Zhang , A. Sapin-Minet , C. Gaucher , and X. Xia , Acta Biomater. 103, 259–271 (2020). 10.1016/j.actbio.2019.12.016 31846803

